# Lifestyle Program for Breast Cancer Improves Body Composition, Fitness, and Patient-Reported Outcomes: A Randomized Clinical Trial †

**DOI:** 10.3390/cancers18111757

**Published:** 2026-05-27

**Authors:** Catherine Powers-James, Aimee J. Christie, Banu Arun, Taylor Austin, Gildy Babiera, Karen Basen-Engquist, Cindy L. Carmack, Alejandro Chaoul, Lisa Connelly Newton, Robin Haddad, Carol Harrison, Cheuk Hong Leung, Yisheng Li, Smitha Mallaiah, Raghuram Nagarathna, Patricia A. Parker, George H. Perkins, Amy Spelman, Anil K. Sood, Richard W. Wagner, Peiying Yang, Sai-Ching J. Yeung, Lorenzo Cohen

**Affiliations:** Unit 1414, Department of Palliative, Rehabilitation, and Integrative Medicine, The University of Texas MD Anderson Cancer Center, 1515 Holcombe Blvd., Houston, TX 77030, USA; cpowers2@mdanderson.org (C.P.-J.); ajchristie@mdanderson.org (A.J.C.); barun@mdanderson.org (B.A.); gvbabiera@mdanderson.org (G.B.); kbasenen@mdanderson.org (K.B.-E.); ccarmack@mdanderson.org (C.L.C.); alechaoul@gmail.com (A.C.); lisamc1210@gmail.com (L.C.N.); rhaddad@alumni.rice.edu (R.H.); caharrison@mdanderson.org (C.H.); cleung@mdanderson.org (C.H.L.); ysli@mdanderson.org (Y.L.); sgmallaiah@mdanderson.org (S.M.); rnagaratna@gmail.com (R.N.); parkerp@mskcc.org (P.A.P.); gperkins@mdanderson.org (G.H.P.); aspelman@mdanderson.org (A.S.); asood@mdanderson.org (A.K.S.); rwagner1@mdanderson.org (R.W.W.); pyang@mdanderson.org (P.Y.); syeung@mdanderson.org (S.-C.J.Y.)

**Keywords:** breast cancer, survivorship, lifestyle, nutrition, physical activity, stress management, radiotherapy, quality of life, integrative medicine

## Abstract

**Importance:** Lifestyle and supportive care interventions may improve treatment tolerance, long-term health behaviors, and survivorship outcomes among breast cancer patients receiving radiotherapy. Few randomized trials have integrated nutrition, exercise, stress management, and psychosocial counseling concurrently with radiotherapy and extended across survivorship. **Objective:** To evaluate the effects of a comprehensive lifestyle intervention (CompLife) initiated during radiotherapy and maintained for 12 months on body composition, fitness, quality of life, symptoms, and nutrition. **Design, Setting, and Participants:** Randomized clinical trial at a single cancer center. Eligible women were aged ≥18 years with stage II/III breast cancer scheduled for radiotherapy, BMI ≥ 24.5, and ≥2 lifestyle risk factors. Participants were randomized to CompLife or standard of care (SOC). Assessments occurred at baseline, end of radiotherapy, and 3, 6, and 12 months. The primary outcome of recurrence is ongoing; this report examines prespecified secondary outcomes. **Interventions:** CompLife included 6 weeks of in-person counseling integrating nutrition, physical activity, mindfulness, stress management, and psychosocial support; twice-weekly exercise, diet, and mind–body sessions during radiotherapy; and telehealth counseling for 12 months. SOC participants received standard educational materials. **Main Outcomes and Measures:** Secondary outcomes included visceral adipose tissue (VAT), weight, waist circumference, fitness (VO_2_ max, strength), SF-36 Physical and Mental Component Summary scores, MD Anderson Symptom Inventory scores, mindfulness (FFMQ), and dietary fiber intake. **Results:** Ninety-five women were randomized (CompLife, n = 50; SOC, n = 45). CompLife participants had lower VAT at 3 and 6 months (e.g., 3 months: 118 vs. 141 cm^2^; *p* = 0.044) and greater improvements in VO_2_ max at 3 (23.4 vs. 18.8 mL/kg/min; *p* = 0.025) and 6 months. SF-36 Physical scores improved to within population norms for CompLife at all follow-ups but remained below norms in SOC. CompLife participants reported fewer symptoms at 12 months (MDASI: 1.3 vs. 2.5; *p* = 0.014). Fiber intake was consistently higher in CompLife participants (20.5 g vs. 14.0 g; *p* < 0.001). The results should be interpreted with caution due to the large number of comparisons among the secondary outcomes. **Conclusions and Relevance:** A multidisciplinary lifestyle intervention delivered during radiotherapy and extended across survivorship produced clinically meaningful improvements in body composition, fitness, diet, and patient-reported outcomes. The findings suggest potential value in integrating structured lifestyle and psychosocial counseling into oncology care and warrant confirmation in larger multicenter trials with mature recurrence endpoints.

## Simple Summary

Lifestyle factors have been linked to better symptom control and clinical outcomes for women undergoing treatment for breast cancer. However, there are limited comprehensive programs that support patients during and after treatment. The current study examined the benefits of a comprehensive program that included healthy eating, exercise, stress management, and emotional and behavioral support (starting during radiation and continuing for a year) on anthropometrics, cardiorespiratory fitness, fiber intake, and quality of life outcomes in women with breast cancer. The findings reveal that this approach may benefit patients in multiple areas, suggesting the value of incorporating structured comprehensive lifestyle programs into standard care. The paper highlights the importance of studying whole-person interventions and supports the need for larger studies to confirm these promising results.

## 1. Introduction

Breast cancer is the most common cancer and the leading cause of cancer-related mortality in women aged 20–59, accounting for 15% of all cancer-related deaths [1]. In 2025, an estimated 316,950 new cases of invasive breast cancer were expected, representing 32% of all new cancer cases among women [2,3]. The incidence is rising by 1% annually, with faster growth among women under 50.

Epidemiological research links lifestyle factors to breast cancer recurrence, disease progression, and mortality [4]. An unhealthy weight, poor diet, physical inactivity, and sedentary behavior are associated with worse outcomes [5,6,7,8]. Specifically, waist circumference and visceral fat are linked to poorer prognoses [9]. Several trials have addressed lifestyle modification. The Women’s Healthy Eating and Living (WHEL) study showed that participants consuming ≥5 servings of fruits/vegetables daily and exercising ≥30 min six days/week had a 50% reduction in mortality [10,11,12]. The Women’s Intervention Nutrition study (WINS) found a 24% reduction in recurrence with a low-fat diet and modest weight loss [13].

Psychosocial factors (e.g., stress, social support, mind–body practices, mindfulness) also affect outcomes [14,15,16,17]. Evidence suggests that psychosocial interventions can influence biological processes related to cancer, such as inflammation and immune function, potentially improving survival [18,19,20,21,22,23]. However, few clinical trials examine comprehensive lifestyle interventions incorporating both behavioral and psychosocial components.

Long-term adherence to healthy behaviors remains a key challenge. Many interventions emphasize diet and exercise but neglect stress-related factors, which may perpetuate unhealthy behaviors [16,24,25]. In contrast, Andersen et al. demonstrated that psychosocial support during chemotherapy improved health behaviors and immune function and reduced recurrence, mortality, and psychological distress [18,26,27,28].

A recent randomized trial using a telehealth lifestyle intervention for weight loss in 3180 women with stage II or III breast cancer with a body mass index (BMI) of 27 or higher reported an average of 4.3 kg (4.7% of baseline body weight) weight loss, whereas the control group gained 0.9 kg. Younger women and Black women did not benefit as much from the program, suggesting that more support may be needed [29].

The current study evaluated a novel, comprehensive lifestyle intervention (CompLife) integrating nutrition, physical activity, mindfulness, stress management, and psychosocial counseling during radiotherapy and across 12 months post-treatment, using a randomized controlled trial (RCT) design [30]. While the primary outcomes—time to cancer recurrence and survival—are ongoing, this paper reports selective secondary outcomes including behavior change, anthropometric measures, fitness, QOL and psychosocial functioning, and diet.

## 2. Methods

### 2.1. Participants

Participants were recruited from the breast radiotherapy center prior to radiation therapy (XRT). Eligibility criteria included age ≥ 18; stage II or III breast cancer scheduled for 4–6 weeks of XRT; body mass index (BMI) ≥ 24.5; oriented to person/place/time; cleared for exercise via physician or according to the Physical Activity Readiness Questionnaire; and at least two lifestyle risk factors (i.e., low fruit/vegetable intake, insufficient physical activity, or infrequent mind–body practice). Exclusions included recurrent/other cancers within 5 years, major thought disorders, communication barriers, uncontrolled diabetes, or severe mobility issues. The original protocol included only women with stage III disease, and this was expanded to stage II to address challenges with recruitment. The study requested that patients make significant lifestyle changes during a stressful period—specifically the start of XRT. Issues such as fatigue, a lack of available time, and focusing on other aspects of their cancer care reduced patient interest in participating in the study.

### 2.2. Procedures

This study was conducted in accordance with the Declaration of Helsinki, the protocol was approved by the MD Anderson Institutional Review Board (Protocol 2012-0112) on 3 March 2014, and informed consent was obtained from all participants. Patients were recruited between 2013 and 2018. The procedures, comprehensive assessment model, intervention, and patient feedback have been detailed previously [30]. Briefly, baseline assessments were conducted prior to randomization and the initiation of radiotherapy and included electronic questionnaires, dual X-ray absorptiometry (DEXA) scans, anthropometrics, fitness testing (including EKG), and dietary recalls. Adaptive randomization, with minimization, was used for the following factors: BMI (dichotomized on a running mean); diabetes (yes/no); age (dichotomized on a running mean); time since diagnosis (dichotomized on a running mean); menopausal status (pre/post); staging; surgery type (segmental/mastectomy); and hormone receptor/HER2/neu status. Randomization was conducted through a central database and staff did not have access to the sequence. Data will be made available to authorized individuals who have human subject approvals from their home institutions. Study personnel were not blinded to group assignment.

Follow-up assessments were conducted at the end of radiotherapy (excluding DEXA) and at 3, 6, and 12 months. To support participation, transportation costs were reimbursed, and participants received up to USD 100 in gift cards per time point for completing the assessments.

### 2.3. CompLife Intervention

The CompLife intervention program was an intensive 4–6-week intervention (determined by whether the women were undergoing four or six weeks of radiotherapy). Previously described [30], it included four main components: (1) 6 weekly counseling sessions integrating motivational interviewing, cognitive behavioral therapy, and psycho-education to facilitate stress management and behavior changes; (2) 12 exercise sessions (twice weekly) during XRT; (3) 12 nutrition sessions, including experiential cooking classes; (4) 12 mind–body sessions, including yoga-based movement, guided meditation, and relaxation practices. The timeline was modified for the women who had only 4 weeks of XRT.

Participants then had weekly telehealth counseling sessions through week 26 and then monthly sessions for the subsequent 6 months (30 sessions maximum) and 2 h booster lifestyle sessions at each follow-up assessment session. They were also provided with a home practice toolkit (e.g., yoga mat, blender, cookbooks, workbook, instructional video and audio recordings, and a copy of the book *Anticancer: A New Way of Life: A New Way of Life* by David Servan-Schreiber) and, for one year, they were provided with a family gym membership and weekly personal training via a community fitness coach.

### 2.4. Standard of Care (SOC)

Participants in the SOC group received general cancer prevention education materials including information on diet, exercise, and stress management and regular contact for data coordination, but no lifestyle counseling. Lifestyle behaviors were monitored to assess potential contamination from outside interventions. This group was not treated as a waitlist control due to the primary outcomes including survival.

### 2.5. Adherence

As reported previously [30], we developed online behavior tracking tools that allowed us to track adherence and used gaming-based theories to compensate patients for the time that they spent tracking their behaviors.

### 2.6. Quality Control

To ensure safety and fidelity, mind–body and resistance training sessions were videotaped for visual and audio review, while counseling and diet sessions were audio-recorded. Ten percent of each interventionist’s sessions were randomly selected for review.

### 2.7. Measures

Demographics were assessed with a demographics questionnaire at baseline, as well as a chart review (e.g., age, BMI, stage, hormone +/−, surgery, ethnicity, race). Key representative measures from the comprehensive assessments are presented, and all represent secondary outcomes.

### 2.8. Anthropometric Outcomes

Anthropometric outcomes included weight, BMI, waist circumference (WC), fat mass index (FMI), and visceral adipose tissue (VAT). BMI was calculated as weight (lb)/[height (in)]^2^ × 703. VAT and FMI were assessed using whole-body dual DEXA scans, a sensitive measure for body composition [31].

### 2.9. Quality of Life Measures

(1) The Medical Outcomes Study 36-Item Short-Form Survey (SF-36) [32] assessed general quality of life. The Physical Health Component Score (PCS) and the Mental Health Component Score (MCS) are reported here, with component scores having a population mean of 50 and SD of 10 [33,34].
(2) The Five Facets of Mindfulness Questionnaire (FFMQ) [35] is a 39-item self-report questionnaire derived from an exploratory factor analysis that measured the tendency to be mindful in everyday life, and the total score is reported [36].
(3) The MD Anderson Symptom Inventory (MDASI) [37] is a multi-symptom self-report measure that assesses the severity of oncology patients’ symptoms (13 symptom items) and their interference with daily living (6 interference items).

### 2.10. Physical Activity Measures

(4) The Godin Leisure-Time Exercise Questionnaire [38,39] is a 4-item self-report questionnaire assessing the number of times that the participant engages in mild (minimal effort), moderate (not exhausting), and strenuous (heart beats rapidly) leisure time activity for at least 15 min in a 7-day period, yielding a total leisure activity score.
(5) Physical Fitness Tests: An exercise physiologist performed the following tests:
  - Arm Curl Test—Upper body strength was assessed with a 30 s arm curl to measure the number of curls completed by each arm with a 5-pound weight.
  - Sit-to-Stand—The 30 s sit-to-stand test examined the number of times that the participant could come to a complete stand within 30 s.
  - Maximal Cardiovascular Capacity—Participants conducted a treadmill test using a modified Balke protocol to calculate the Metabolic Equivalent of Task (METS). Breath-by-breath and 30 s averaging measurements of VO_2_ (ml/kg/min), METs, and caloric expenditure (kcal/min) were ascertained throughout the test using the ParvoMedics True One metabolic measurement cart (Parvo Medics, Salt Lake City, UT, USA). These data were used to estimate the functional maximal cardiovascular capacity, expressed as VO_2_ max.

### 2.11. Dietary Measure

Dietary Recall—Three 24 h dietary recalls on three randomly assigned non-consecutive days (two weekdays and one weekend day) over a 2-week period at each time point were conducted using a standardized protocol to calculate fiber content as the primary dietary outcome [40]. Only fiber is reported to reduce the number of variables assessed, as it reflects fruit and vegetable consumption and is relevant to microbiome biodiversity.

### 2.12. Statistical Analyses

The trial was powered for the primary outcome of recurrence of disease or death with 80 patients in each group, with a total number of events required of 60, to detect differences in recurrence-free survival at 4 years of 50% and 70% between the two groups, with a 1-sided significance level of 0.05 and 80% power, assuming no dropouts before 4 years. At 5 and 10 years, these rates are estimated to be 42% and 64% and 18% and 41%, respectively. Due to slow accrual, recruitment was stopped at N = 100. As a result, we acknowledge the inadequate power to detect the originally targeted treatment effect on the primary outcome of recurrence-free survival. Group differences in baseline characteristics were assessed using two-sample *t*-tests (age) and chi-squared (ethnicity) or Fisher’s exact tests (race, stage, hormone, surgery). Linear mixed models (LMMs) compared secondary outcomes at post-treatment and follow-ups (3, 6, 12 months), controlling for baseline values. Within-group changes were also examined. We did not impute missing data, assuming that data were missing at random, in which case the maximum likelihood estimates in LMMs are valid without missing data imputation. Analyses were performed in SAS 9.4. As the measures included in this study are secondary endpoints of the trial and no multiple testing adjustments were made, and results need to be interpreted as hypothesis-generating.

## 3. Results

**Figure 1:**
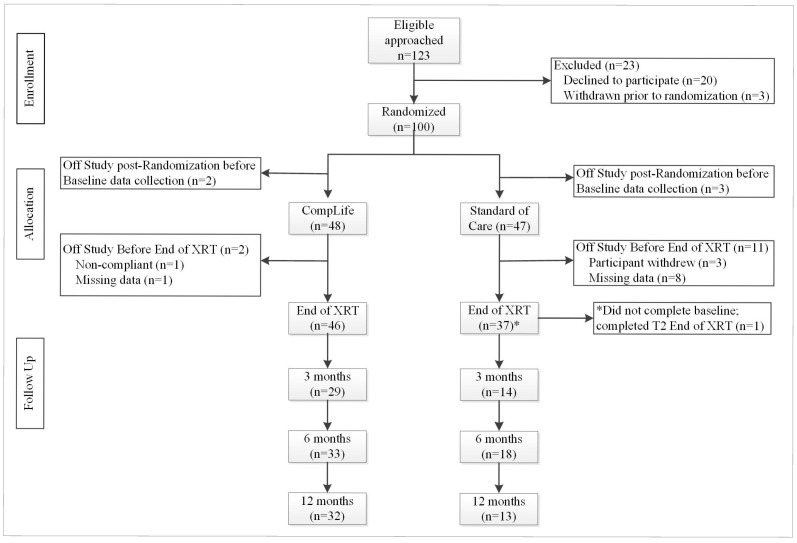
CONSORT Diagram.

Of the 123 eligible patients approached for participation (see CONSORT Figure 1), 20 declined to participate, two did not meet the inclusion criteria after providing consent, and one did not provide baseline data. Four more patients dropped out after randomization and before data collection, with a total of 95 participants enrolled in the study (enrolled/eligible = 83%); CompLife n = 50 and SOC n = 45. Two participants were excluded from VAT analyses due to machine malfunction. There was a varying amount of attrition over time, with more dropouts in the SOC group (12 months: CompLife n = 32; SOC n = 13). Patients did not indicate reasons for attrition and were simply lost to follow-up. Sociodemographic and outcome data are presented in Table 1. At baseline, there were no significant group differences between CompLife and SOC. On average, women were 49 years old (SD = 10.9). Seventy-five percent of participants were non-Hispanic and 71% were White. The BMI for both groups was 31 at baseline.

Intervention adherence was high, with 78% of participants missing three or fewer sessions. By the end of radiation, adherence to behavioral prescriptions was robust: all participants met dietary and physical activity goals, and 67% met the mind–body practice targets. Daily self-report tracking corroborated these outcomes. At baseline, none of the participants reported engaging in meditation; however, by the end of XRT, 75% practiced for ≥30 min per day, with an average duration of 35 min (range 30–80 min). Regarding dietary changes, 60% of participants exceeded the target ratio of 90:10 for health-supporting to health-depleting foods, 30% met the goal, and 20% achieved a ratio of 80:20.

Physical activity adherence was similarly strong, with 75% of participants exercising for ≥30 min per day and 15% exceeding 60 min daily. Retention and continued counseling participation post-treatment were also high: 26% of participants were fully compliant with all post-XRT sessions, and 80% attended at least half. Four participants in the intervention arm and four in the standard-of-care group withdrew during the follow-up period.

### 3.1. Anthropometric Measures

Table 2 shows group comparisons over time. When comparing groups over the course of treatment, CompLife participants had a significantly lower WC than the SOC group at each time point. CompLife participants also had a significantly lower weight, FMI, and BMI than the SOC group at 3, 6, and 12 months and lower VAT at 3 and 6 months. Within-group changes from baseline revealed decreases in all outcomes for CompLife and increases for SOC.

### 3.2. Quality of Life Measures

Table 3 shows the group comparisons over time for psychosocial outcomes. The SF-36 PCS was significantly higher at each follow-up time point for CompLife participants versus SOC. CompLife participants had clinically significant improvements (five points or greater) [41], with scores being below the population means at baseline and significantly improving from baseline at each time point and being within the population means. In contrast, SOC participants’ PCS scores remained below the population means at each time point, with small but significant improvements from baseline at only 3 and 6 months. There were no group differences for the SF-36 MCS, with mean scores being within the population mean at each time point (50, SD = 10) [42]. However, significant within-group improvements from baseline for CompLife were apparent at 3, 6, and 12 months and only at 12 months for SOC.

The FFMQ revealed that the CompLife participants had higher scores than the SOC group at the 3-month time point, as well as showing a greater improvement from baseline than the SOC group. Significant within-group improvements were noted for CompLife participants from baseline to each time point, with no significant changes in the SOC group.

The MDASI Symptom subscale showed that CompLife participants had significantly improved symptom severity by 12 months compared to SOC participants. The MDASI Interference subscale showed that CompLife participants had lower scores following XRT but not at other time points, as well as within-group changes at the end of XRT and at 3 and 12 months, while there were no changes over time for the SOC group for either subscale.

### 3.3. Physical Activity Measures

Godin scores revealed statistically significant improvements in activity levels at 3, 6, and 12 months for CompLife participants compared to SOC participants (Table 4). Within-group analyses revealed that both the SOC and CompLife groups improved on the Godin score after XRT, but only the CompLife group continued to maintain improvements at 3, 6, and 12 months. Similarly, improvements for both right and left arm curls were significantly different at the end of XRT and 6 and 12 months for the CompLife participants relative to SOC, with improvements in both groups over time compared to baseline. The sit-to-stand test, VO_2_ max, and total METS showed that CompLife participants had greater improvements at 3 and 6 months compared to SOC, with no improvements over time for SOC.

### 3.4. Dietary Measures

Mean fiber intake was significantly higher at each time point for CompLife participants than SOC (Table 5), with consistent increases from baseline in CompLife and decreases from baseline in fiber for SOC.

## 4. Discussion

The present study evaluated a comprehensive lifestyle intervention in breast cancer patients. Prior studies typically address nutrition, exercise, or stress individually but rarely integrate all three domains. Our program successfully combined the three components and showed that CompLife was associated with better anthropometrics, improved fitness, and increased fiber intake compared to SOC. It also led to improved quality of life and mindfulness and reduced symptom burdens. Notably, while SOC participants worsened over time in anthropometric indices, CompLife participants maintained improvements. These findings suggest that comprehensive long-term interventions may be effective at improving multiple patient outcomes.

Although CompLife participants showed benefits in all domains, there was variability in the timing of between-group differences. Several group differences were only seen by 3 months and then continued into the maintenance phase of CompLife, such as with the Godin score, weight, and BMI. This reflects not only the importance of tracking changes over extended periods of time but also of the importance of long-term interventions. Previously, we reported qualitative feedback from several women in the program [43]; one theme emphasized patients’ desire for long-term support for lifestyle changes and stress management. Thus, not only do patients desire more long-term lifestyle change assistance, but, by tracking changes over the course of a year, we can better understand health benefits over time.

Other between-group differences were only seen at the earlier time points and were not maintained over 12 months, as seen with METS, VO_2_ max, and sit-to-stand. It is possible that, immediately after finishing the program, patients are more motivated to engage in lifestyle change. It is also possible that patients have more time to focus on their health immediately after radiation treatment but, later in the year, typical life demands, such as work and family commitments, make it more difficult for patients to continue with their lifestyle change goals.

The results of the present study are comparable to the outcomes of a psychosocial intervention designed by Anderson and colleagues [18], which showed improvements in health behaviors, quality of life, and psychological distress when using mental health providers to facilitate groups. We aimed to expand on this study by incorporating the expertise of multiple professionals in the following domains: exercise (physical therapist), nutrition (registered dietitian), mind–body practices (yoga therapist), and mental health (licensed counselor).

A notable strength of this study was that the intervention was initiated while participants were in treatment and continued for 12 months. Not only have patients expressed a preference for long-term comprehensive lifestyle programs [43], but the American College of Cardiology/American Heart Association’s task force recommends comprehensive lifestyle modification programs that include at least 14 visits over 6 months and trained interventionists that focus on nutrition, physical activity, and behavioral strategies [44]. CompLife was designed to offer the maximum intervention support based on the premise that psychological well-being and healthy lifestyle choices have bidirectional relationships. As reported previously, patients noted that stress management skills and mind–body practices were critically important to facilitate healthy habits in the areas of diet and exercise [30]. However, the mental health scores as assessed with the MCS indicated that the women were doing well at each time point and within the population means, which suggests that perhaps the stress management component was linked with behavior changes and less with mental health per se. Future studies using this sample can examine mediators and moderators of behavior change, as well as the survival outcomes.

## 5. Limitations

This study was conducted at a single clinical center and focused exclusively on women with stage II or III disease, limiting the generalizability to other cancer populations. The majority (78%) of the study sample was White. Although this is comparable to the percentage of White patients served at the MD Anderson Cancer Center (76.5%) [45], it is unclear if CompLife meets all the needs of patients of color. For example, a culturally tailored mobile app health intervention improved diet and physical activity behaviors in African Americans [46]. In lifestyle change programs, men are also often underrepresented [4]. To account for this limitation, future studies may consider expanding similar programs to other cancer diagnoses and underserved groups, as well as emphasizing the recruitment of men.

Another limitation of the study was participant attrition, particularly within the SOC group. By the 12-month follow-up, the outcome with the most data (DEXA) covered 32 patients in CompLife and 13 in SOC, and only 27 participants (19 in CompLife group and 8 in SOC group) provided questionnaire responses. It is unclear if patients dropped out due to disengagement from the CompLife recommendations, the burden of repeated extensive assessments, or other unrelated reasons. Patients with barriers such as working full time, caring for family members, or difficulty commuting to the medical center may have dropped out at a higher rate. The attrition in the current study highlights the need for research efforts to focus on maintaining patient engagement and various options for data collection. The high attrition over time, especially in the SOC group, limits the generalizability of our findings and introduces potential bias in long-term outcome interpretation, particularly if missing data were not random. However, the higher attrition rate in the control group for the long-term follow-ups may suggest potential biases in the direction of underestimating the intervention’s effects. Additionally, as personnel were not blinded to group assignment, there is the potential for assessment bias. Finally, as the results from this study are secondary outcomes, they are exploratory in nature and are intended to be hypothesis-generating. Due to the large number of comparisons, it is possible that some results are positive due to chance.

## 6. Conclusions

This study demonstrates that a comprehensive lifestyle intervention can yield meaningful improvements in anthropometric, quality of life, physical fitness, and dietary measures among women with stage II and III breast cancer undergoing radiotherapy and throughout the following year. Given the observed benefits of the CompLife intervention and the existing research in this area, oncology providers are encouraged to engage patients in discussions about evidence-based lifestyle changes, including nutrition, physical activity, and stress management, as a component of cancer care and how this may benefit patients. Future studies should aim to expand the applicability of these findings across a broader range of oncology populations, as well as including patients with significant psychosocial stressors and higher levels of obesity. They should also explore strategies to sustain and improve long-term adherence to comprehensive lifestyle interventions.

## Figures and Tables

**Table 1:** Baseline demographic and medical characteristics.

Baseline Characteristics		Overall n (%)	CompLife n (%)	SOC n (%)	*p*-Value
		95 (100)	50 (53)	45 (47)	
Age	Mean (SD)	49.3 (10.9)	49.3 (10.0)	49.3 (11.9)	0.98
Race	Asian	4 (4)	2 (4)	2 (4)	0.62
	Black (African American)	18 (19)	12 (24)	6 (13)	
	White	67 (71)	33 (66)	34 (76)	
	Others	6 (6)	3 (6)	3 (7)	
Ethnicity	Hispanic	24 (25)	13 (26)	11 (24)	0.86
	Not Hispanic	71 (75)	37 (74)	34 (76)	
Stage	IIA	8 (8)	4 (8)	4 (9)	0.92
	IIB	14 (15)	8 (16)	6 (13)	
	IIIA	35 (37)	19 (38)	16 (36)	
	IIIB	8 (8)	3 (6)	5 (11)	
	IIIC	30 (32)	16 (32)	14 (31)	
Hormone	ER+ PR+ HER+	11 (12)	5 (10)	6 (13)	0.99
	ER+ PR+ HER−	52 (55)	27 (54)	25 (56)	
	ER+ PR − HER+	2 (2)	1 (2)	1 (2)	
	ER+ PR − HER−	5 (5)	3 (6)	2 (4)	
	ER − PR − HER+	8 (8)	4 (8)	4 (9)	
	ER − PR − HER−	17 (18)	10 (20)	7 (16)	
Surgery	Bilateral mastectomy with reconstruction	12 (13)	6 (12)	6 (13)	0.87
	Bilateral mastectomy without reconstruction	2 (2)	1 (2)	1 (2)	
	Breast-conserving surgery	12 (13)	5 (10)	7 (16)	
	Breast-conserving surgery with reconstruction	1 (1)	1 (2)	0 (0)	
	Unilateral mastectomy with reconstruction	35 (37)	18 (36)	17 (38)	
	Unilateral mastectomy without reconstruction	33 (35)	19 (38)	14 (31)	
Radiation	Four weeks	12 (13)	4 (8)	8 (17)	0.20
	Six weeks	83 (87)	44 (92)	39 (83)	

**Table 2:** Anthropometric measures at baseline and least square means adjusted for baseline at end of XRT, 3 months, 6 months, and 12 months.

		Mean (SE)			
Variable	Timepoint	CompLife	SOC	Estimate (SE) *	*p*-Value
Weight (kg)	Baseline	79.9 (1.9)	80.8 (1.9)		
	End of XRT	78.9 (1.9)	81.1 (1.9)	−1.17 (0.9)	00.19
	3 months	77.7 (1.9) ^b^	81.5 (2.0)	−2.86 (1.2)	00.02
	6 months	78.6 (1.9)	82.7 (2.0) ^a^	−3.34 (1.2)	00.004
	12 months	79.1 (1.9)	83.5 (2.1) ^b^	−3.55 (1.3)	00.005
BMI	Baseline	31.0 (0.7)	31.4 (0.7)		
	End of XRT	30.4 (0.7) ^a^	31.6 (0.7)	−0.61 (0.3)	00.07
	3 months	30.2 (0.7) ^b^	31.8 (0.8)	−1.12 (0.5)	00.02
	6 months	30.5 (0.7)	32.1 (0.8) ^a^	−1.14 (0.4)	00.009
	12 months	30.8 (0.7)	32.5 (0.8) ^b^	−1.19 (0.5)	00.01
Waist (cm)	Baseline	91.7 (1.5)	92.5 (1.5)		
	End of XRT	89.9 (1.5) ^c^	93.5 (1.5)	−2.84 (0.8)	<0.001
	3 months	90.1 (1.5) ^b^	93.4 (1.6)	−2.45 (1.1)	00.03
	6 months	89.8 (1.5) ^b^	93.9 (1.6)	−3.41 (1.00	<0.001
	12 months	90.0 (1.5) ^b^	94.3 (1.7)	−3.67 (1.1)	00.001
FMI	Baseline	13.8 (0.5)	14.0 (0.5)		
	-				
	3 months	13.0 (0.5) ^c^	14.3 (0.5)	−1.18 (0.4)	00.005
	6 months	13.2 (0.5) ^a^	14.8 (0.5) ^b^	−1.24 (0.4)	00.002
	12 months	13.7 (0.5)	15.2 (0.6) ^c^	−1.32 (0.4)	00.002
VAT	Baseline	126 (8.0)	137 (8.3)		
	-				
	3 months	118 (8.6)	141 (9.6)	−15.8 (7.8)	00.04
	6 months	123 (8.5)	150 (9.4) ^a^	−16.0 (7.5)	00.04
	12 months	126 (8.5)	148 (9.9)	−11.9 (7.9)	00.14

Note: BMI = body mass index, (lb)/[height (in)]^2^ × 703; FMI = fat mass index; VAT = visceral adipose tissue. * Least square mean estimates (SE) and *p*-values are for between-group comparisons at each time point using mixed model analyses, covarying for baseline values. Baseline values are raw means, and follow-up time points are the least square mean estimates adjusted for baseline. Within-group statistically significant differences between baseline and follow-up time points are indicated by the following superscripts: ^a^ = *p* ≤ 0.05; ^b^ = *p* ≤ 0.01; ^c^ = *p* ≤ 0.001.

**Table 3:** Patient-reported outcomes at baseline and least square means adjusted for baseline at end of XRT, 3 months, 6 months, and 12 months for CompLife and SOC groups.

		Mean (SE)			
Variable	Timepoint	CompLife	SOC	Estimate (SE) *	*p*-Value
SF-36 (PCS)	Baseline	37.6 (1.3)	38.7 (1.3)		
	End of XRT	46.0 (1.5) ^c^	39.8 (1.7)	6.74 (2.0)	0.001
	3 months	51.8 (1.6) ^c^	43.3 (1.9) ^a^	8.96 (2.3)	<0.001
	6 months	49.9 (1.7) ^c^	43.6 (2.0) ^a^	6.83 (2.4)	0.005
	12 months	51.0 (1.7) ^c^	42.0 (2.5)	9.32 (2.7)	<0.001
SF-36 (MCS)	Baseline	48.3 (1.6)	45.6 (1.7)		
	End of XRT	51.9 (1.9)	46.0 (2.2)	4.12 (2.7)	0.129
	3 months	52.5 (2.0) ^a^	49.1 (2.5)	2.03 (2.9)	0.492
	6 months	54.0 (2.2) ^b^	48.8 (2.6)	3.86 (3.1)	0.217
	12 months	53.3 (2.1) ^a^	53.8 (3.2) ^b^	−2.25 (3.5)	0.522
MDASI (Symptoms)	Baseline	2.0 (0.2)	2.1 (0.2)		
	End of XRT	1.5 (0.3) ^a^	2.1 (0.3)	−0.51 (0.3)	0.11
	3 months	1.6 (0.3)	2.2 (0.3)	−0.50 (0.4)	0.15
	6 months	1.8 (0.3)	2.0 (0.3)	−0.23 (0.4)	0.54
	12 months	1.3 (0.3) ^b^	2.5 (0.4)	−1.08 (0.4)	0.01
MDASI (Interference)	Baseline	2.3 (0.3)	2.6 (0.3)		
	End of XRT	1.5 (0.4) ^a^	2.9 (0.5)	−1.25 (0.5)	0.02
	3 months	1.2 (0.4) ^b^	1.8 (0.5)	−0.55 (0.6)	0.36
	6 months	1.8 (0.5)	1.9 (0.5)	0.05 (0.7)	0.94
	12 months	1.2 (0.5) ^b^	2.6 (0.7)	−1.24 (0.8)	0.10
FFMQ	Baseline	137.3 (2.7)	139.0 (2.8)		
	End of XRT	145.2 (3.2) ^b^	138.4 (3.6)	7.82 (4.2)	0.07
	3 months	149.2 (3.3) ^c^	137.9 (3.8)	12.64 (4.5)	0.006
	6 months	146.1 (3.6) ^b^	142.9 (4.0)	4.50 (4.8)	0.36
	12 months	147.5 (3.5) ^c^	143.3 (4.8)	5.65 (5.5)	0.31

Note: SF-36 (PCS) = Medical Outcomes Study 36-Item Short-Form Survey Physical Health Component Score; SF-36 (MCS) = Medical Outcomes Study 36-Item Short-Form Survey Mental Health Component Score; MDASI = MD Anderson Symptom Inventory; FFMQ = Five Facets Mindfulness Questionnaire. * Least square mean estimates (SE) and *p*-values are from the between-group mixed model analyses at each time point, covarying for baseline values. Baseline values are raw means, and follow-up time points are the least square mean estimates adjusted for baseline. Within-group statistically significant differences between baseline and follow-up time points are indicated by the following superscripts: ^a^ = *p* ≤ 0.05; ^b^ = *p* ≤ 0.01; ^c^ = *p* ≤ 0.001.

**Table 4:** Fitness measures at baseline and estimated least square means adjusted for baseline at end of XRT, 3 months, 6 months, and 12 months.

		Mean (SE)			
Variable	Timepoint	CompLife	SOC	Estimate (SE) *	*p*-Value
Godin	Baseline	15.4 (4.7)	12.3 (4.8)		
	End of XRT	55.2 (6.1) ^c^	33.9 (6.8) ^b^	18.96 (11.2)	0.093
	3 months	65.9 (6.7) ^c^	27.7 (7.9)	36.92 (12.5)	0.004
	6 months	54.2 (7.9) ^c^	23.9 (8.2)	28.67 (13.7)	0.04
	12 months	52.9 (7.1) ^c^	9.1 (10.6)	43.28 (15.3)	0.006
Right Arm Curl	Baseline	16.5 (0.7)	15.1 (0.7)		
	End of XRT	19.3 (0.7) ^c^	16.4 (0.8) ^a^	1.64 (0.8)	0.047
	3 months	19.5 (0.8) ^c^	16.8 (1.0) ^a^	1.28 (1.1)	0.23
	6 months	21.0 (0.8) ^c^	17.7 (0.9) ^c^	2.22 (1.0)	0.03
	12 months	21.5 (0.8) ^c^	18.0 (1.0) ^c^	2.21 (1.1)	0.046
Left Arm Curl	Baseline	15.9 (0.7)	15.7 (0.7)		
	End of XRT	19.0 (0.7) ^c^	16.9 (0.8) ^a^	1.91 (0.8)	0.02
	3 months	19.8 (0.8) ^c^	17.9 (1.0) ^a^	1.52 (1.1)	0.16
	6 months	21.1 (0.8) ^c^	18.1 (0.9) ^b^	2.97 (1.0)	0.004
	12 months	21.6 (0.8) ^c^	18.8 (1.0) ^c^	2.66 (1.1)	0.02
Sit-to-Stand	Baseline	12.9 (0.5)	12.8 (0.5)		
	End of XRT	14.3 (0.5) ^c^	13.2 (0.6)	0.91 (0.5)	0.09
	3 months	15.2 (0.6) ^c^	12.8 (0.7)	2.15 (0.7)	0.002
	6 months	15.1 (0.6) ^c^	12.9 (0.7)	2.08 (0.7)	0.002
	12 months	15.0 (0.6) ^c^	13.5 (0.7)	1.27 (0.7)	0.08
VO_2_ max— Relative (mL/kg/min)	Baseline	21.1 (0.8)	18.8 (0.8)		
	End of XRT	22.5 (0.8) ^a^	19.5 (0.8)	1.17 (0.9)	0.19
	3 months	23.4 (0.9) ^c^	18.8 (1.2)	3.03 (1.3)	0.03
	6 months	24.6 (0.8) ^c^	20.2 (1.2)	2.61 (1.2)	0.03
	12 months	22.1 (0.9)	19.5 (1.4)	0.52 (1.5)	0.73
METS (kcal/min)	Baseline	6.02 (0.2)	5.39 (0.2)		
	End of XRT	6.43 (0.2) ^a^	5.57 (0.2)	0.33 (0.2)	0.15
	3 months	6.65 (0.2) ^c^	5.37 (0.3)	0.84 (0.3)	0.02
	6 months	7.01 (0.2) ^c^	5.76 (0.3)	0.72 (0.3)	0.02
	12 months	6.54 (0.2) ^b^	5.59 (0.4)	0.37 (0.4)	0.33

Note: VO_2_ max = maximum volume of oxygen; METS = Metabolic Equivalent of Task. * Least square mean estimates (SE) and *p*-values are from the between-group mixed model analyses at each time point, covarying for baseline values. Baseline values are raw means, and follow-up time points are the least square mean estimates adjusted for baseline. Within-group statistically significant differences between baseline and follow-up time points are indicated by the following superscripts: ^a^ = *p* ≤ 0.05; ^b^ = *p* ≤ 0.01; ^c^ = *p* ≤ 0.001.

**Table 5:** Fiber intake at baseline and least square means adjusted for baseline at end of XRT, 3 months, 6 months, and 12 months.

		Mean (SE)			
Variable	Timepoint	CompLife	SOC	Estimate (SE) *	*p*-Value *
Fiber	Baseline	17.2 (1.2)	16.9 (1.2)		
	End of XRT	22.7 (1.2) ^c^	14.0 (1.2) ^a^	8.29 (1.7)	<0.001
	3 months	20.3 (1.3) ^b^	15.1 (1.5)	5.63 (1.9)	0.003
	6 months	21.6 (1.3) ^c^	14.2 (1.6)	7.36 (2.0)	<0.001
	12 months	20.5 (1.3) ^b^	14.0 (1.6)	6.80 (2.0)	<0.001

* Least square mean estimates (SE) and *p*-values are from the between-group mixed model analyses at each time point, covarying for baseline values. Baseline values are raw means, and follow-up time points are the least square mean estimates adjusted for baseline. Within-group statistically significant differences between baseline and end of XRT, 3 months, 6 months, and 12 months are indicated by the following superscripts: ^a^ = *p* ≤ 0.05; ^b^ = *p* ≤ 0.01; ^c^ = *p* ≤ 0.0.

## Data Availability

The data presented in this study are available on request from the corresponding author. Data will be made available to authorized individuals who have human subjects approvals from their home institutions.
